# New Therapeutic Concept of NAD Redox Balance for Cisplatin Nephrotoxicity

**DOI:** 10.1155/2016/4048390

**Published:** 2016-01-05

**Authors:** Gi-Su Oh, Hyung-Jin Kim, AiHua Shen, Su-Bin Lee, Sei-Hoon Yang, Hyeok Shim, Eun-Young Cho, Kang-Beom Kwon, Tae Hwan Kwak, Hong-Seob So

**Affiliations:** ^1^Center for Metabolic Function Regulation, Department of Microbiology, School of Medicine, Wonkwang University, Iksan, Jeonbuk 570-749, Republic of Korea; ^2^Department of Internal Medicine, School of Medicine, Wonkwang University, Iksan, Jeonbuk 570-749, Republic of Korea; ^3^Department of Oriental Medical Physiology, College of Korean Medicine, Wonkwang University, Iksan, Jeonbuk 570-749, Republic of Korea; ^4^PAEAN Biotechnology, 160 Tachno-2 Street, Yuseong-gu, Daejeon 305-500, Republic of Korea

## Abstract

Cisplatin is a widely used chemotherapeutic agent for the treatment of various tumors. In addition to its antitumor activity, cisplatin affects normal cells and may induce adverse effects such as ototoxicity, nephrotoxicity, and peripheral neuropathy. Various mechanisms such as DNA adduct formation, mitochondrial dysfunction, oxidative stress, and inflammatory responses are closely associated with cisplatin-induced nephrotoxicity; however, the precise mechanism remains unclear. The cofactor nicotinamide adenine dinucleotide (NAD^+^) has emerged as a key regulator of cellular energy metabolism and homeostasis. Recent studies have demonstrated associations between disturbance in intracellular NAD^+^ levels and clinical progression of various diseases through the production of reactive oxygen species and inflammation. Furthermore, we demonstrated that reduction of the intracellular NAD^+^/NADH ratio is critically involved in cisplatin-induced kidney damage through inflammation and oxidative stress and that increase of the cellular NAD^+^/NADH ratio suppresses cisplatin-induced kidney damage by modulation of potential damage mediators such as oxidative stress and inflammatory responses. In this review, we describe the role of NAD^+^ metabolism in cisplatin-induced nephrotoxicity and discuss a potential strategy for the prevention or treatment of cisplatin-induced adverse effects with a particular focus on NAD^+^-dependent cellular pathways.

## 1. Introduction

*cis*-Diamminedichloroplatinum II (CDDP, cisplatin) is a widely used chemotherapeutic drug for the treatment of various solid tumors in the head and neck, bladder, lung, ovaries, testicles, and uterus [1–6]. The various adverse effects of cisplatin during the course of chemotherapy include ototoxicity, nephrotoxicity, myelosuppression, and peripheral neuropathy. Cisplatin accumulates in renal tissues and cells, which are primary sites for drug filtration, concentration, and excretion. Even if blood concentrations are held at nontoxic levels during chemotherapy, concentrations may reach toxic levels in the kidneys [4]. In general, cisplatin concentrations in tubular epithelial cells of kidney tissues are five times higher than those in blood, and the elevated concentration of cisplatin therein causes nephrotoxicity. Clinical signs of kidney damage are a decrease in renal plasma flow and glomerular filtration rate, an increase of serum creatinine and blood urea nitrogen, and a reduction of serum magnesium and potassium levels [7]. Cisplatin-induced nephrotoxicity is dose-dependent and therefore limits the potential to increase dosage for optimal cancer therapy [8]. Even though the establishment of cisplatin-induced nephrotoxicity can be alleviated by diuretics and prehydration of patients, the prevalence of cisplatin nephrotoxicity is still high, occurring in approximately one-third of patients who have undergone cisplatin therapy [6]. Cisplatin nephrotoxicity can present in a number of ways, including acute kidney injury, hypomagnesemia, hypocalcemia, hyperuricemia, distal renal tubular acidosis, proximal tubular dysfunction, and chronic renal failure [7]. However, the most serious and one of the more common clinical features of cisplatin nephrotoxicity is acute kidney injury which occurs in 20–30% of patients. Recent studies have demonstrated that the cellular process of nephrotoxicity can be attributed to local accumulation of cisplatin inside the proximal tubule by membrane transportation, and intracellular conversion of the drug into toxic metabolites. Furthermore, various mechanisms such as DNA adduct formation, mitochondrial dysfunction, oxidative stress, inflammatory responses, and activation of apoptotic pathways are closely associated with cisplatin-induced nephrotoxicity [7].

The cofactor nicotinamide adenine dinucleotide (NAD^+^) has emerged as a key regulator of cellular energy metabolism and homeostasis. Recently, it has been reported that intracellular NAD^+^/NADH ratios are decreased in various pathological conditions such as diabetes [9], cisplatin-induced cochlear and kidney damage [10, 11], and in many tissues of aged animals and humans [12, 13]. Recent studies have demonstrated that a disturbance in intracellular NAD^+^ levels is linked to the progression of various diseases through the production of reactive oxygen species (ROS) and inflammation [10, 11, 14]. Furthermore, reduction of the intracellular NAD^+^/NADH ratio is critically involved in cisplatin-induced acute kidney damage; increasing the cellular NAD^+^/NADH ratio by pharmacological agents suppresses cisplatin-induced acute kidney damage by downregulation of potential damage mediators such as oxidative stress and inflammatory responses [11]. The decrease in the NAD^+^/NADH ratio has been attributed to hyperactivation of the NAD^+^-consuming poly(ADP-ribose) polymerase 1 (PARP-1) induced by oxidative damage due to altered redox mechanisms and consequent DNA damage [10, 11]. Since silent mating type information regulation 2 homolog 1 (sirtuin 1, SIRT1) deacetylase activity is influenced by the NAD^+^/NADH ratio [15], a significant reduction in the NAD^+^/NADH ratio causes a concomitant decrease in SIRT1 deacetylase activity, which is critically involved in diverse biological functions. In addition, the *α*-ketoglutarate dehydrogenase (*α*-KGDH) complex, an enzyme complex of the Krebs cycle in mitochondria, facilitates the generation of ROS after NAD^+^/NADH reduction [16]. Decreased NAD^+^/NADH also favors ROS generation in the respiratory chain complex I [17]. Therefore, maintenance of adequate NAD^+^ levels may be a critical factor for normal cellular function and could emerge as a useful strategy for treating many diseases.

Although there are review articles each focusing on cisplatin-mediated nephrotoxicity or beneficial role of NAD^+^, there is lack of effort illustrating a potential therapeutic or preventive strategy of modulatory NAD^+^ levels for treating cisplatin-associated nephrotoxicity. Therefore, we aimed to review a critical issue related to cisplatin-induced nephrotoxicity which can potentially be overcome by modulation of cellular NAD^+^ levels. We searched PubMed for published articles using separate search terms “cisplatin-mediated nephrotoxicity” and “NAD^+^-modulation and disease” and included only most recent and relevant publication including original research articles and reviews but excluded repetitive illustrations. In this review, we describe the mechanisms of cisplatin-mediated nephrotoxicity and the role of NAD^+^ metabolism therein and discuss a potential strategy for prevention of the adverse effects of cisplatin through targeting of NAD^+^-dependent cellular pathways.

## 2. Kidney-Specific Toxicity of Cisplatin: Cisplatin Transport and Biotransformation

Cisplatin is primarily cleared by the kidneys through both glomerular filtration and tubular secretion, whereas the biliary and intestinal excretions of this drug are negligible. During the excretion process, cisplatin is highly concentrated in the kidneys, which suggests an active accumulation of this drug by renal parenchymal cells, thereby explaining the particular damage caused by this drug to the kidneys compared to other organs. The toxic effects of cisplatin occur primarily in the renal proximal tubules, predominantly in the epithelial tubular cells of the S-3 segment [18]. Though the high concentrations of cisplatin in the kidneys favor its cellular uptake by passive diffusion, recent studies have demonstrated two different membrane transporters capable of facilitating the transport of cisplatin into cells. Copper transporter 1 (Ctr1) is highly expressed and localized in the basolateral membrane of the proximal tubule in the adult kidney [19]. Even though the role of Ctr1 in cisplatin nephrotoxicity* in vivo* has not been examined, cisplatin uptake and cytotoxicity were decreased by downregulation of Ctr1 expression in kidney cells* in vitro*, suggesting that Ctr1 is required for cisplatin uptake in these cells. In addition, the organic cation transporter 2 (OCT2) is specifically expressed in the basolateral membranes of the kidney renal proximal tubule cells, contributing to the etiology of the organ-specific toxicity of cisplatin. OCT2 expression is critical for the development of cisplatin-induced nephrotoxicity, and introduction of the OCT2 substrate cimetidine, a competing factor for transport, reduces nephrotoxicity [20], suggesting that OCT2 is critically involved in cisplatin uptake and its toxicity in these cells. In addition to specific expression of cisplatin transporter proteins in the kidney, many studies have demonstrated that cisplatin undergoes metabolic activation in the kidney to a more potent nephrotoxin. This process is initiated with the biotransformation of cisplatin-glutathione (GSH) conjugates by glutathione-S-transferase in the circulation [21]. When the cisplatin-GSH conjugate reaches and passes through the kidney, it is cleaved to a nephrotoxic metabolite primarily by the action of gamma-glutamyl transpeptidase, an enzyme principally located on the surface of the kidney proximal tubule cells. This metabolite is a highly reactive thiol/platinum compound that interacts with macromolecules, eventually leading to renal damage [22].

## 3. Mechanisms of Cisplatin Nephrotoxicity

### 3.1. Oxidative Stress in Cisplatin Nephrotoxicity

Oxidative stresses, including superoxide anions, hydrogen peroxide, and hydroxyl radicals, are unavoidable by-products of cellular respiration. Oxidative stress is also closely involved in renal injury after cisplatin administration. In particular, production of ROS and antioxidant system dysfunction are associated with cisplatin-induced nephrotoxicity [23]. Due to their unstable and highly reactive nature, ROS may attack and modify multiple target molecules such as lipids, proteins, and DNA, producing cellular stress. ROS also activate important signaling pathways, including an apoptotic pathway, which leads to cell death in the event of cisplatin-induced nephrotoxicity [24]. Although the role of oxidative stress in renal damage is well established, its source is poorly understood. Potential sources of ROS include the mitochondrial electron transport chain system [25], xanthine oxidase [26], cytochrome P450 enzymes [27], and NADPH oxidase [28]. Cisplatin may produce ROS in microsomes via the cytochrome P450 system (CYP).* In vitro* and* in vivo* tests have demonstrated that CYP is an important source of catalytic iron for the generation of ROS during cisplatin treatment. Furthermore, the cisplatin-induced increase of ROS and kidney damage were attenuated in CYP2E1^−/−^ mice [29]. Mitochondria have also been reported to be a major source of ROS. Disturbance of the mitochondrial electron transport chain system, which was accompanied by loss of mitochondrial membrane potential, an indicator of mitochondrial dysfunction, is a well-recognized mechanism responsible for the generation of ROS [25]. Interestingly, mitochondria themselves are particularly vulnerable to oxidative stress. Oxidative damage to mitochondria causes the impairment of mitochondrial function and subsequent cell death via apoptosis and necrosis [30]. Thus, ROS-mediated oxidative damage to mitochondria favors the generation of additional ROS, resulting in a vicious cycle. Many studies have demonstrated a clear association between mitochondrial ROS generation and cisplatin nephrotoxicity [6]. Membrane NAD(P)H oxidases (NOXs) are also one of the major sources for ROS generation. Especially in phagocytic cells such as neutrophils, superoxide is generated by NOX enzyme complexes [31]. However, many studies have recently found that superoxide-generating NOX expression is not restricted to phagocytic cells but is present in a wide variety of nonphagocytic cells and tissues [32]. In particular, it has been reported that superoxide-generating NOXs are expressed in the inner ear and in kidney tissues and that their expression is increased by exposure to cisplatin, thereby causing oxidative stress that leads to cisplatin-mediated ototoxicity and nephrotoxicity [11, 33, 34].

### 3.2. Inflammation in Cisplatin Nephrotoxicity

In addition to direct cellular toxicity, inflammation is closely associated with the pathogenesis of cisplatin nephrotoxicity. Over the last decade, it has been found that a number of mediators of inflammation, including TNF-*α*, IL-1*β*, TGF-*β*, RANTES, MIP2, and MCP1, are increased in cisplatin-induced renal injury. Inflammation contributes to the development of renal tissue damage and renal failure under pathological conditions. However, evidence for a functional role in renal damage for many of these cytokines still remains to be identified, with the exception of TNF-*α* [35, 36]. The proinflammatory cytokine TNF-*α* plays a central role in many infectious and inflammatory diseases. Relevantly, the functional involvement of TNF-*α* in the pathogenesis of cisplatin-induced acute renal failure was determined in mice treated with cisplatin in the presence or absence of TNF-*α* production inhibitors, as well as in TNF-*α* knockout mice. Treatment with TNF-*α* production inhibitors reduced cisplatin-induced renal damage and also reduced histologic evidence of injury. TNF-deficient mice were also resistant to cisplatin nephrotoxicity. These results indicated an important role for TNF-*α* in the pathogenesis of cisplatin nephrotoxicity [37]. Furthermore, this study showed that pharmacological inhibitors and antibodies against TNF-*α* markedly suppressed the induction of other cytokines during cisplatin nephrotoxicity, suggesting that TNF-*α* might be a key upstream regulator of the inflammatory response triggered by cisplatin. These observations have been confirmed and extended by other studies [38–40]. TNF-*α* can be produced by a variety of both immune and nonimmune cells. However, Zhang et al. were able to determine the source of the TNF-*α* that was responsible for cisplatin-induced renal damage [39]. They created chimeric mice in which TNF-*α* could be produced by resident kidney cells or by circulating immune cells and evaluated kidney function, histology, and cytokine expression in these chimeric mice following cisplatin administration. In this study, they demonstrated that the local production of TNF-*α* by resident kidney cells, probably the renal epithelial cells themselves, was crucial to cisplatin-induced nephrotoxicity [39].

The next question, then, became: how does TNF-*α* stimulate the inflammatory response and contribute to cisplatin nephrotoxicity? The biological activities of TNF-*α* are primarily mediated by two functionally distinct receptors, TNFR1 and TNFR2, to induce a variety of cellular responses ranging from inflammation to cell death. TNFR1 and TNFR2 are also upregulated by cisplatin. While TNFR1 directly induces the extrinsic apoptotic pathway, TNFR2 is primarily associated with the inflammatory response, which amplifies the TNFR1 effects. Furthermore, as the TNFR2 protein does not contain the death domain necessary to trigger apoptosis, TNFR2, unlike TNFR1, would appear to indirectly induce apoptosis and necrosis in renal tubular epithelial cells [41, 42]. Conflictingly, Tsuruya et al. showed that TNFR1-deficient mice and renal tubular cells were more resistant to cisplatin-induced renal injury and apoptosis compared with wild type mice [43], whereas Ramesh and Reeves recently showed that cisplatin-induced tubular cell death and renal injury were clearly attenuated in TNFR2-deficient, but not in TNFR1-deficient, mice [41]. Although the cause of the inconsistency between these two studies has not been elucidated, together they suggest that TNF-*α* signaling plays a critical role for cisplatin nephrotoxicity. The production of TNF-*α* after cisplatin administration is highly dependent upon ROS, NF-*κ*B activation, and activation of p38 MAPK. In fact, TNF-*α* both is an inducer of ROS and is induced by ROS generated by cisplatin [37]. ROS activates the transcription factor NF-*κ*B, which in turn induces the production of proinflammatory cytokines such as TNF-*α* [42]. NF-*κ*B activation is pivotal in the expression of proinflammatory cytokines and other mediators involved in acute inflammatory responses and other conditions associated with increased ROS generation [44]. In addition to direct oxidative damage to lipids, DNA, and proteins [23], ROS generated by cisplatin activates p38 MAPK through the induction of p38 MAPK phosphorylation, which mediates the synthesis of TNF-*α*. Ramesh and Reeves demonstrated that inhibition of p38 MAPK reduced TNF-*α* production and protected against cisplatin-induced renal damage* in vivo* [45]. Activation of p38 MAPK led to the degradation of I*κ*B (an inhibitor of NF-*κ*B), thereby promoting translocation of NF-*κ*B to the nucleus and the consequent stimulation of proinflammatory cytokine production, including TNF-*α* [46].

Toll-like receptors (TLRs) are a family of pattern recognition receptors that detect pathogenic elements such as viral RNA, bacterial DNA, lipopolysaccharides, or proteins, called pathogen-associated molecular patterns (PAMPs). TLRs play a pivotal role in host defense against infection by sensing the invasion of organisms and initiating both innate and adaptive immune responses [47]. TLRs also detect and respond to certain endogenous molecules such as high-mobility group box protein 1 (HMGB1), heat shock proteins (HSPs), and extracellular matrix components, termed damage-associated molecular pattern molecules (DAMPs). DAMPs are generally released by damaged or stressed tissues to “alert” the immune system to tissue injury or impending danger [48]. Cisplatin increases the expression of TLRs, including TLR4 in murine peritoneal macrophages* in vitro*, and subsequent stimulation by individual TLR-related ligands induces the production of proinflammatory cytokines such as TNF-*α*, IFN-*γ*, IL-1*β*, and IL-12 [49]. In addition, Zhang et al. have demonstrated that TLR4 is essential to the initiation of intrarenal inflammatory cytokine production associated with cisplatin-induced nephrotoxicity [50]. Ramesh et al. also have demonstrated that the combination of cisplatin and lipopolysaccharides, which are specific ligands for TLR4, acts synergistically to produce inflammatory cytokines such as TNF-*α*, IL-6, MCP-1, KC, and GM-CSF, thereby inducing nephrotoxicity in an acute renal failure model [51].

NF-*κ*B activation is a critical bridge to the expression of inflammatory cytokines and other mediators involved in inflammatory responses through TLR signaling. After dimerization of TLR4 through engagement with its ligand, adapter molecules such as TIRAP and TRAM are recruited on the cytoplasmic domain of TLR4, which further interacts with MyD88 and TRIF, respectively, and then transduces a signal to the nucleus. MyD88 is critical for signaling by all TLRs except TLR3. After stimulation, MyD88 associates with the cytoplasmic portion of the TLR and recruits IL-1R-associated kinase- (IRAK-) 4 and -1 through a homophilic interaction of the death domains. Subsequently, TRAF6, TAK1, and NF-*κ*B are activated, and then NF-*κ*B is translocated into the nucleus where it regulates the genes for proinflammatory cytokines among others [52]. In contrast, in TLR4 knockout mice, the activation of p38, which is critical for cisplatin-induced TNF-*α* production, was significantly blunted [45]. Finally, the released nuclear protein HMGB1 has been shown to activate TLR4 in various pathologic conditions [48, 53]; however, the role of HMGB1 and other DAMPs in TLR4 activation associated with cisplatin nephrotoxicity remains to be elucidated.

### 3.3. Role of NAD Redox Balance in Cisplatin Nephrotoxicity

NAD is a metabolic cofactor that is present in cells either in its oxidized (NAD^+^) or in its reduced (NADH) form. NAD^+^ or NADH functions as a cofactor for a multitude of enzymatic reactions and therefore critically regulates cellular energy metabolism and homeostasis. As NAD^+^ is critical for a variety of enzymatic reactions, including glycolysis, the NAD redox balance, represented as the NAD^+^/NADH ratio, is tightly regulated [54], and its disruption has been associated with multiple clinical disorders and pathologies. For example, pellagra is caused by NAD^+^ deficiency subsequent to poor dietary intake of NAD^+^ biosynthesis precursors and can be easily cured by providing dietary nicotinic acid [55]. Pathological conditions such as diabetes and oxidative stress are also well correlated with decreased cellular NAD^+^ levels [9, 56]. It has also been recently reported that the cellular NAD^+^ level in many tissues declines with age [12, 13], implying the importance of maintaining optimal intracellular NAD^+^ levels to prevent age-associated cellular dysfunction. Furthermore, cisplatin-induced cochlear and kidney damage are highly associated with the decreases of NAD^+^/NADH ratios that accompany inflammation and oxidative stress [10, 11]. Cisplatin treatment resulted in a decrease of NAD^+^/NADH ratio in renal tissue without significant changes of NADH level [11]. This suggests that the decrease of NAD^+^/NADH ratio by cisplatin is mainly caused by reduction of NAD^+^ level. Of note, *β*-lapachone coadministration with cisplatin also restored NAD^+^/NADH ratio to control level through elevation of NAD^+^ level, but not by decrease of NADH level. Together, these findings suggest that maintenance of the NAD redox balance is very important for general health.

### 3.4. Role of NAD^+^-Dependent Enzymes in Cisplatin Nephrotoxicity

NAD^+^ acts as a cofactor for numerous enzymes including SIRTs, PARPs, and cyclic ADP- (cADP-) ribose synthases [57]; therefore, NAD^+^ might exert its biological effect through these enzymes. The mammalian sirtuin family consists of seven enzymes, SIRT1–7 [58], that are ubiquitously expressed yet show specific cellular localizations and functions. SIRT1, SIRT6, and SIRT7 are generally localized in the nuclei of cells, whereas SIRT3, SIRT4, and SIRT5 are localized in the mitochondria [59]. SIRT1 and SIRT5 act exclusively as deacetylases [60, 61], whereas SIRT2, SIRT3, SIRT4, and SIRT6 might also have a mono-ADP-ribosyl transferase activity [60, 62–64]. SIRT1 is the most widely studied sirtuin and has a* Km* for NAD^+^ that lies within the range of the physiological changes in intracellular NAD^+^ content. This suggests that sirtuin activity could be modulated by the physiological changes in intracellular NAD^+^ levels [64]. Considering that the intracellular NAD^+^/NADH ratios are decreased in various pathological conditions, including cisplatin-induced nephrotoxicity and ototoxicity [9–11, 56], SIRT1 activity might be reduced in the damaged tissues as well. In particular, Hasegawa et al. demonstrated that SIRT1 protects against oxidative stress-induced apoptosis in the kidney by inducing catalase, which catalyzes the decomposition of the ROS hydrogen peroxide, via deacetylation of FOXO3 in cultured proximal tubular cells [65]. Furthermore, Hasegawa et al. also reported that renal proximal tubular cell-specific* SIRT1* transgenic mice showed resistance to cisplatin-induced renal tubular cell injuries such as apoptosis by maintaining peroxisome number and function, concomitant with upregulation of catalase and elimination of renal ROS [66]. In addition, it has been demonstrated that SIRT1 activation by resveratrol reduced cisplatin-induced proximal tubular cell apoptosis through deacetylation of p53 [67]. In contrast, Kim et al. and Oh et al. demonstrated that the reduction of intracellular NAD^+^/NADH ratio in cisplatin-injected kidney and cochlear tissues was critically associated with the decline of SIRT1 activity, which thereby caused cisplatin-induced nephrotoxicity and ototoxicity through inflammation and oxidative stress [10, 11]. However, SIRT1 activation through the increase of the cellular NAD^+^/NADH ratio suppressed the adverse effects of cisplatin by downregulation of potential damage mediators such as oxidative stress factors and inflammatory responses.

SIRT1 regulates diverse biological functions through direct interaction with and subsequent deacetylation of its targets, including p53 and NF-*κ*B, which are closely related to its function in cisplatin-induced nephrotoxicity [7]. As described previously, the transcription factor NF-*κ*B is one of the key regulators of inflammation. NF-*κ*B activation is attained by either I*κ*B phosphorylation and subsequent degradation or an I*κ*B-independent pathway through posttranslational modifications of the NF-*κ*B Rel proteins, including acetylation of the NF-*κ*B p65 subunit. NF-*κ*B p65 can be acetylated at five specific lysine residues (Lys-122, Lys-123, Lys-218, Lys-221, and Lys-310). In particular, acetylation of the Lys-310 residue is required for the transcriptional activity of NF-*κ*B, whereas the other acetylation sites are involved in DNA binding [68]. SIRT1 physically interacts with the nuclear translocated NF-*κ*B p65 subunit and deacetylates it at Lys-310, thereby inhibiting the transcriptional activity of NF-*κ*B [69]. An assortment of recent evidence indicates that SIRT1 regulates inflammatory response through NF-*κ*B p65 deacetylation. In cisplatin-induced nephrotoxicity and ototoxicity, Kim et al. demonstrated that SIRT1 activation was critically associated with the deacetylation status of the NF-*κ*B p65 subunit [10, 11]. In addition, it has been demonstrated that SIRT1 knockdown leads to inflammatory pathway activation with increased inflammatory gene expression, whereas SIRT1 activation produces anti-inflammatory effects [70].

The tumor suppressor p53 is another crucial transcription factor in the cellular stress response [71]. A number of posttranslational modifications can occur in p53 that have critical effects on its stability and function, including phosphorylation, acetylation, sumoylation, neddylation, and methylation [72]. Cytosolic p53 is bound to Mdm2, a RING finger E3 ubiquitin ligase that facilitates protein degradation under normal conditions. Cellular stress, including DNA damage, hypoxia, or oxidative stress, induces rapid mitochondrial translocation of p53 and its posttranslational modification such as acetylation by p300/CBP or PCAF acetyltransferase [73]. The p53 is acetylated at lysine residues, including Lys-370, Lys-372, Lys-382, and Lys-386 in the carboxy-terminal region. Because acetylated p53 cannot bind to Mdm2, increased p53 acetylation levels strongly correlate with protein stabilization and activation in response to cellular stress [74]. Both nuclear SIRT1 and mitochondrial SIRT3 regulate p53 function through direct interaction and subsequent deacetylation of p53 [75]. In the nucleus, acetylation of p53 stimulates its sequence-specific DNA binding and subsequent recruitment of other transcription cofactors to promoter regions and thereby enhances transcription of target genes [76–78] such as the p53-upregulated modulator of apoptosis (PUMA), NADPH activator A (NOXA), and p53-induced gene 3 (PIG3), all of which are involved in the production of ROS through mitochondrial dysfunction or apoptosis. Deacetylation of p53 by nuclear-localized SIRT1 inactivates its sequence-specific transcriptional activity and represses p53-mediated cell growth arrest and apoptosis in response to DNA damage and oxidative stress [74]. Mitochondrial-localized SIRT3 deacetylates and activates several enzymes that are critical in maintaining cellular ROS levels and for apoptosis. Though it is not well understood whether acetylated p53 in mitochondria might have other functions, mitochondrial p53 interacts with anti- and proapoptotic Bcl-2 family members to either inhibit or activate them, thereby promoting apoptosis through robust mitochondrial outer membrane permeabilization and subsequent cytochrome c release [79, 80]. Deacetylation of p53 by mitochondrial-localized SIRT3 also represses p53-mediated cell growth arrest and apoptosis in response to DNA damage and oxidative stress [75]. On the other hand, Kim et al. demonstrated that cisplatin treatment led to substantial elevation of acetylated p53 levels in the kidney and cochlear tissues compared to those of untreated normal control mice [10, 11].

NAD^+^ is consumed not only by sirtuins, but also by PARPs [81]. Cisplatin accumulation in target tissues produces ROS that deplete the cellular antioxidant defense factors necessary to reduce oxidative stress and DNA damage. Cisplatin also directly binds to DNA, resulting in the disruption of the synthesis of key proteins and leading to cell injury and cell death. Furthermore, accumulation of DNA damage can lead to cell cycle arrest or genomic instability. The removal of DNA damaged by oxidative stress is mediated by single-strand DNA break repair, which is facilitated by PARPs. PARP-1 is the most critical protein-modifying nuclear enzyme involved in DNA repair. PARP-1 is a major NAD^+^ consumer, wherein the ADP-ribose moiety is transferred to PARP-1 itself or to other acceptor proteins in order to build the poly(ADP-ribose) polymer (PAR) [82]. PARP-1 is strongly activated by DNA damage and oxidative stress. Under physiological conditions, mild activation of PARP-1 can regulate several cellular processes, including DNA repair, cell cycle progression, cell survival, chromatin remodeling, and genomic stability [83]. However, hyperactivation of PARP-1 upon severe oxidative damage causes rapid depletion of intracellular NAD^+^ and ATP levels and eventually leads to cell death and related pathological conditions [84, 85]. Kim et al. have demonstrated that hyperactivation of PARP-1 in cisplatin-treated cochlea led to a decline in intracellular NAD^+^ levels and SIRT1 activity, thereby causing cochlear damage [10]. It is well established that PARP-1 and SIRT1 activity are interdependent as they compete for a limited pool of cellular NAD^+^. However, the* Km* of PARP-1 for NAD^+^ is two to ten times lower than that of SIRT1, which falls within the physiological range of cellular NAD^+^ concentrations [86]. Thus, PAPR-1 activation might critically influence SIRT1 activity by reducing NAD^+^ bioavailability. This model was further supported by recent studies wherein genetic depletion of PARP-1 or pharmacological inhibition of PARP-1 activity increased intracellular NAD^+^ level and subsequent SIRT1 activity [10].

## 4. Therapeutic Considerations of Cisplatin Nephrotoxicity

The main protective actions currently employed in clinical practice to reduce nephrotoxicity during cisplatin chemotherapy are based on avoiding the extreme exposure of the kidneys to the drug. This is managed primarily by hydration/diuretics, monitoring of renal function by serum creatinine clearance, and decreasing cisplatin doses upon manifestation of renal dysfunction [87, 88]. However, even with aggressive hydration, renal toxicity occurs. Therefore, more effective preventative strategies without attenuation of tumoricidal activity need to be developed, taking into consideration the mechanisms underlying the adverse effects of cisplatin exposure. Although the exact mechanism responsible for cisplatin-associated cellular damage is still to be elucidated, numerous studies have indicated that ROS and increased inflammation are important factors. The roles of these two factors seem to be closely related, and thus their abnormal regulation impacts overlapping cellular processes. Accordingly, pharmacological interventions that reduce systemic inflammation and/or oxidative stress might prevent or alleviate the development and progression of cisplatin-induced nephrotoxicity. However, these effects need to be explored* in vivo* [7]. A better option might be to focus on maintaining a proper level of intracellular NAD^+^. The decrease of cellular NAD^+^ level in the kidney and cochlear tissues after cisplatin exposure [10, 11] implies the therapeutic potential of intracellular NAD^+^ level modulation for cisplatin-associated adverse effects. The role of NAD^+^ in the prevention and cure of diseases was first recognized in the 1930s by Conrad Elvehjem, who demonstrated the therapeutic effect of the vitamin nicotinic acid on pellagra in dogs [55]. Since then, the therapeutic potential of NAD^+^ has been further evidenced by several studies. Araki et al. showed that addition of exogenous NAD^+^ to neurons delayed axonal degeneration in response to mechanical or chemical damage [89]. Ying et al. also demonstrated that intranasal administration of NAD^+^ profoundly decreased brain injury in a rat model of transient focal ischemia [90]. Pillai et al. showed that exogenous NAD^+^ blocked cardiac hypertrophic response [91].

**Figure 1:**
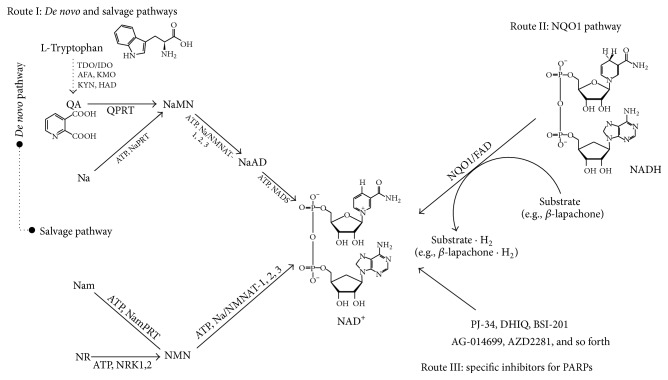
Possible pathways for mammalian NAD^+^ biosynthesis. The biosynthesis of NAD^+^ through* de novo*, salvage, NQO1 pathways, and specific inhibition for PARPs. ATP: adenosine triphosphate, FAD: flavin adenine dinucleotide, IDO: indoleamine 2,3-dioxygenase, Na: nicotinic acid, NaAD: nicotinic acid adenine dinucleotide, NAD: nicotinamide adenine dinucleotide, NADS: NAD synthetase, Nam: nicotinamide, NaMN: nicotinic acid mononucleotide, NaPRT: nicotinic acid phosphoribosyl transferase, NMN: nicotinamide mononucleotide, NMNAT: nicotinamide mononucleotide adenylyltransferase, NQO1: NAD(P)H:quinone oxidoreductase 1, NR: nicotinamide riboside, NRK1,2: nicotinamide riboside kinase 1, 2, NamPRT: nicotinamide phosphoribosyltransferase, NMNAT: nicotinamide mononucleotide adenyltransferase, QA: quinolinic acid, QPRT: quinolinate phosphoribosyltransferase, TDO: tryptophan 2,3-dioxygenase, AFA: arylformamidase, KMO: kynurenine 3-monooxygenase, KYN: kynureninase, HAD: 3-hydroxy-anthranilate 3,4-dioxygenase, and PARPs: poly(ADP-ribose) polymerases.

As NAD^+^ regulates SIRTs that are involved in various cellular processes, the beneficial effects observed following enhanced SIRT activity might be attributed to increased intracellular NAD^+^ levels. Since both PARPs and SIRTs are NAD^+^-consuming enzymes and thus compete for NAD^+^, selective blockage of NAD^+^-consuming PARPs might also potentially be a good strategy to increase NAD^+^ levels. Consistent with this notion, targeted PARP inactivation has been shown to increase NAD^+^ levels and increase SIRT1 activity [92], suggesting that the modulation of PARP activity could be a therapeutic strategy for the treatment of cisplatin-associated adverse effects. In addition, approaches aimed at increasing NAD^+^ levels by supplementing NAD^+^ precursors through the activation of* de novo* and salvage pathways (Figure 1) for NAD^+^ biosynthesis have demonstrated cytoprotective effects against cellular damage. In fact, this specific strategy has been shown to increase NAD^+^ levels both* in vitro* and* in vivo*. For example, the administration of nicotinamide, a NAD^+^ precursor, showed a protective effect against oxidative stress and glucose deprivation* in vitro* and also alleviated tissue damage in animal models of ischemia [93, 94], spinal cord injury [95], and multiple sclerosis [96]. Similarly, nicotinic acid, another NAD^+^ precursor, has also been used to treat hyperlipidemia [97], indicating the therapeutic potential of NAD^+^ precursors. Although NAD^+^ treatment has not been tested extensively for its cytoprotective effects, a recent report suggested that it might reduce brain damage by protecting against PARP-1-induced cell death [98, 99].

**Figure 2:**
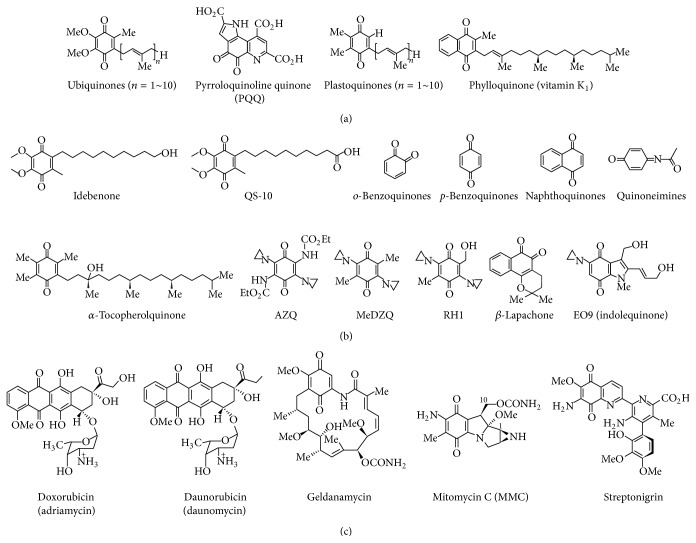
Substrates for NQO1. (a) Naturally occurring quinones as antioxidants. (b) Substrates for reduction by NQO1 and reactive quinone metabolites (benzoquinone, naphthoquinone, and quinoneimine). (c) Naturally occurring quinones with anticancer properties. QS-10: 6-(9-carboxynonyl)-2,3-dimethoxy-5-methyl-1,4-benzoquinone, AZQ: Diaziquine, MeDZQ: 2,5-diaziridinyl-3,6-dimethyl-1,4-benzoquinone, and RH1: 2,5-diaziridinyl-3-hydroxymethyl-6-methyl-1,4-benzoquinone.

**Figure 3:**
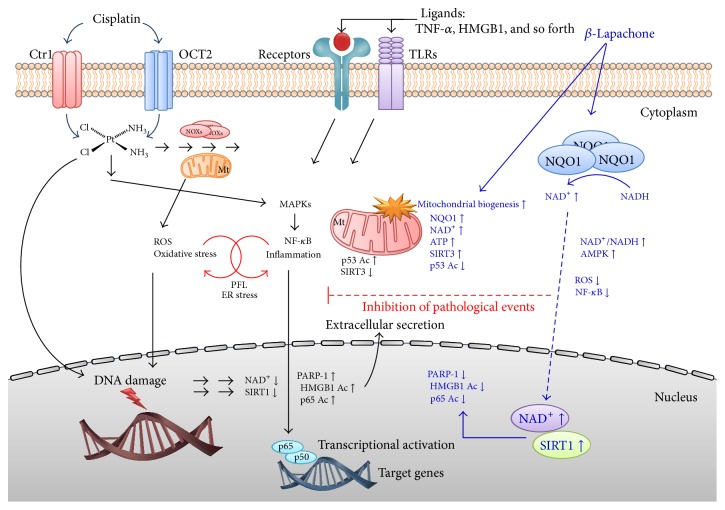
Role of NAD^+^ and NAD^+^-dependent enzymes in cisplatin-induced nephrotoxic mechanisms. Ctr1: copper transporter 1, OCT2: organic cation transporter 2, NOXs: NADPH oxidoreductases, Mt: mitochondria, ROS: reactive oxygen species, PFL: positive feedback loop, ER: endoplasmic reticulum, TLRs: toll-like receptors, HMGB1: high-mobility group box protein 1, TNF-*α*: tumor necrosis factor-*α*, NF-*κ*B: nuclear factor-*κ*B, NQO1: NAD(P)H:quinone oxidoreductase 1, and PARPs: poly(ADP-ribose) polymerases.

As shown in Figure 1, another strategy in addition to* de novo* and salvage NAD^+^ biosynthesis pathways for regulating cellular NAD^+^ levels might be to utilize the cytosolic flavoprotein NADH : quinone oxidoreductase 1 (NQO1) that normally participates in reduction of quinone compounds in exchange for NADH oxidation [100, 101]. NQO1 catalyzes the reduction of quinones to hydroquinones by utilizing NADH as an electron donor, which consequently increases intracellular NAD^+^ levels. Therefore, it is plausible that endogenous factors or chemical agents that potentially activate NQO1 enzymatic activity or act as strong substrates of NQO1 might be beneficial for protection against cisplatin-induced toxicity by increasing intracellular NAD^+^ levels. In addition, there is evidence that NQO1 also plays a role in other biological activities, including anti-inflammatory processes, the scavenging of superoxide anion radicals, and the stabilization of p53 and other tumor suppressor proteins [102–106]. As shown in Figure 2, several substrates of NQO1 enzyme, including mitomycin C, RH1, AZQ, Coenzyme Q10, and idebenone, have been identified [107, 108], of which *β*-lapachone (3,4-dihydro-2,2-dimethyl-2H-naphtho[1,2-b]pyran-5,6-dione) is recently well studied as a strong substrate of NQO1 [109, 110]. *β*-Lapachone was first isolated from the bark of the Lapacho tree and was reported to inhibit tumor growth [111]. Several reports have indicated that pharmacological substrates of NQO1 ameliorate phenotypic manifestations associated with pathological conditions in rodent models. In particular, metabolic diseases such as obesity and spontaneous hypertension were shown to be reversed upon NQO1 enzymatic action using *β*-lapachone [112, 113], and pathological conditions such as arterial restenosis due to tissue injury and cisplatin-associated nephrotoxicity were also ameliorated by NQO1 enzymatic action using this substrate [11, 114]. As summarized in Figure 3, the increase of the cellular NAD^+^/NADH ratio by *β*-lapachone prevents cisplatin-induced kidney damage by modulation of potential damage mediators such as oxidative stress and inflammatory responses. Furthermore, *β*-lapachone did not interfere with the tumoricidal effect of cisplatin* in vivo* [11].

## 5. Conclusion

In conclusion, a NAD redox balance is critically important for sustaining a healthy condition, and maintenance of adequate NAD redox balance may show therapeutic benefits in various diseases through the regulation of NAD^+^-dependent enzymes and their downstream targets including SIRTs, PARPs, NF-*κ*B, and p53. In this review we strongly suggest for the first time that direct modulation of a cellular NAD redox balance by pharmacological agents could be a promising therapeutic approach for the treatment of various diseases, including cisplatin nephrotoxicity.
